# Evaluation of Ultraviolet-C Light for Rapid Decontamination of Airport Security Bins in the Era of SARS-CoV-2

**DOI:** 10.20411/pai.v5i1.373

**Published:** 2020-05-22

**Authors:** Jennifer L. Cadnum, Daniel F. Li, Lucas D. Jones, Sarah N. Redmond, Basya Pearlmutter, Brigid M. Wilson, Curtis J. Donskey

**Affiliations:** 1 Research Service; Louis Stokes Cleveland VA Medical Center; Cleveland, Ohio; 2 Department of Molecular Biology and Microbiology; Case Western Reserve University School of Medicine; Cleveland Ohio; 3 Case Western Reserve University School of Medicine; Cleveland, Ohio; 4 Geriatric Research, Education, and Clinical Center; Louis Stokes Cleveland VA Medical Center; Cleveland, Ohio

**Keywords:** ultraviolet light, SARS-CoV-2, airport, decontamination, fomites

## Abstract

**Background:**

Contaminated surfaces are a potential source for spread of respiratory viruses including SARS-CoV-2. Ultraviolet-C (UV-C) light is effective against RNA and DNA viruses and could be useful for decontamination of high-touch fomites that are shared by multiple users.

**Methods:**

A modification of the American Society for Testing and Materials standard quantitative carrier disk test method (ASTM E-2197-11) was used to examine the effectiveness of UV-C light for rapid decontamination of plastic airport security bins inoculated at 3 sites with methicillin-resistant *Staphylococcus aureus* (MRSA) and bacteriophages MS2, PhiX174, and Phi6, an enveloped RNA virus used as a surrogate for coronaviruses. Reductions of 3 log_10_ on inoculated plastic bins were considered effective for decontamination.

**Results:**

UV-C light administered as 10-, 20-, or 30-second cycles in proximity to a plastic bin reduced contamination on each of the test sites, including vertical and horizontal surfaces. The 30-second cycle met criteria for decontamination of all 3 test sites for all the test organisms except bacteriophage MS2 which was reduced by greater than 2 log_10_ PFU at each site.

**Conclusions:**

UV-C light is an attractive technology for rapid decontamination of airport security bins. Further work is needed to evaluate the utility of UV-C light in real-world settings and to develop methods to provide automated movement of bins through a UV-C decontamination process.

## INTRODUCTION

The emergence of highly pathogenic respiratory viruses such as severe acute respiratory syndrome coronavirus 2 (SARS-CoV-2) highlights the need for effective measures to prevent viral transmission [1]. Respiratory viruses are shed in high concentrations in respiratory secretions [2–4]. Exposure to respiratory droplets and direct contact with contaminated individuals, including hand contact, are important routes of transmission [5]. The importance of contaminated surfaces and fomites in transmission is uncertain [5]. However, respiratory viruses including SARS-CoV-2 can survive for hours to days on surfaces [5–7]. Many studies have demonstrated recovery of respiratory virus nucleic acid on surfaces in healthcare and community settings such as households, day care centers, schools, and airports [5, 8–13]. Based on this body of evidence, enhanced environmental cleaning and disinfection is recommended as a control measure for SARS-CoV-2 in healthcare and community settings [14].

Ultraviolet-C (UV-C) light is effective in killing RNA and DNA viruses [15–20] and is commonly used in healthcare facilities for post-discharge room decontamination as an adjunct to standard cleaning and disinfection [21–22]. UV-C technologies could also potentially be useful for decontamination of high-touch fomites that are shared by multiple users in healthcare or community settings [22]. The efficacy of UV-C light is substantially reduced with increased distance from the light source, on soft versus hard surfaces, and in areas out of the direct line of sight of the UV-C lamp [21–23]. Therefore, ideal fomites for UV-C decontamination would be hard, smooth surfaces that can be placed close to UV-C bulbs allowing short treatment cycles and that are not readily amenable to standard cleaning and disinfection processes that may be equally effective [22].

In the current study, we evaluated the potential for UV-C to be used for rapid decontamination of airport security bins. Security bins were chosen for study because they are repeatedly contacted by people and personal items in a setting with limited potential for alternative rapid decontamination strategies. In addition, recent studies have demonstrated frequent respiratory virus contamination of airport surfaces, including security bins [12–13]. Short UV-C cycles were studied because short exposure can be effective when items are close to the UV-C source [21–22, 24–25], and because decontamination of high-touch items between each use could potentially provide much greater benefit than intermittent decontamination after many uses.

## METHODS

### Test organisms

Table 1 shows the test organisms studied and their characteristics. The enveloped double-stranded RNA virus bacteriophage Phi 6 (Félix d'Hérelle Reference Center for bacterial viruses of the Université Laval HER 102) has been used as a surrogate for coronaviruses and influenza in previous studies [26]. Bacteriophage Phi6 was propagated in *Pseudomonas syringae* as previously described [26]. The bacteriophages MS2 and Phi X174 were propagated in *Escherichia coli* as previously described [27].

**Table 1.** Characteristics of the test organisms

Organism	Source	Characteristics
Methicillin-resistant *Staphylococcus aureus* (MRSA)	Clinical isolate; pulsed-field gel electrophoresis type USA 400	Non-spore-forming Gram-positive bacterium
Bacteriophage Phi X174	ATCC 13706-B1	Nonenveloped single-stranded DNA virus (27 µm particle size)
Bacteriophage Phi6	HER 102	Enveloped, double-stranded RNA virus (85 µm particle size)
Bacteriophage MS2	ATCC 15597-B1	Nonenveloped, single-stranded RNA virus (26 µm particle size)

Abbreviations: ATCC, American Type Culture Collection; HER, Félix d'Hérelle Reference Center for bacterial viruses of the Université Laval.

### Efficacy of UV-C light for decontamination of security bins

**Figure 1.**
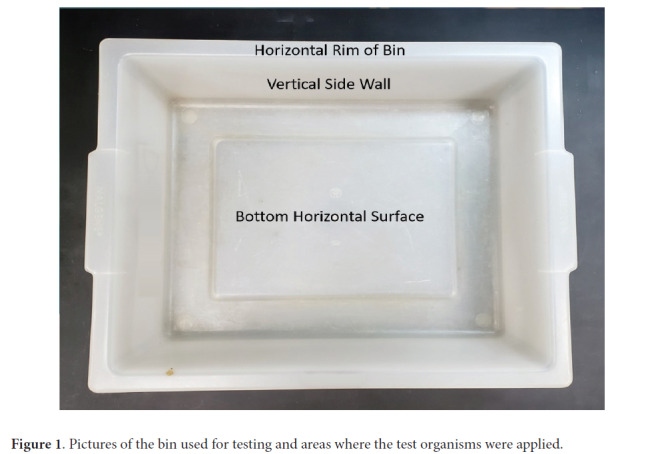
Pictures of the bin used for testing and areas where the test organisms were applied.

Testing was performed using a modification of the American Society for Testing and Materials standard quantitative carrier disk test method (ASTM E-2197-11) [28]. A large variety of different bin types are used in airports. The plastic bins selected for study were autoclave bins that are similar in size and shape to many bins used in airports. The bins were rectangular with length, width, and depth of 54, 43.5, and 13 cm, respectively. For each pathogen, 10-μL aliquots containing 10^6^ CFU or PFU of the test organisms were suspended in 8% simulated mucus [29] and were spread to cover 20-mm diameter circular areas on the bins and allowed to air dry. The inoculated areas included the bottom horizontal surface of the bin, the vertical side wall of the bin, and the horizontal top surface of the rim that is often gripped by hands (Figure 1). Two standard low-pressure mercury UV-C lamps each providing 426 µW/cm^2^ (Philips 30W bulbs, Cambridge, MA) with a target wavelength 254 nm were extended horizontally 7.6 cm apart at a height of 1 inch above the top edge of the bin. The UV-C lamps were operated for 10, 20, or 30 seconds delivering a fluence of 8,520, 17,040, and 25,560 µW/cm^2^ respectively. The short cycle time was chosen based on the presumption that rapid turn-around time would be essential for airport security bins.

After the UV-C treatment, the inoculation sites were sampled using pre-moistened BD Culture-Swabs (Becton Dickinson). The swabs were vortexed for 1 minute in 200 µL phosphate-buffered saline with 0.02% Tween. Serial dilutions were plated on selective media to quantify viable organisms [7, 24, 27]. All tests were performed in triplicate. Similar experiments were conducted with the test organisms inoculated onto 20-mm steel disk carriers, spread to cover the surface area, and air dried. The swabs and disks were processed as previously described, and log_10_CFU or PFU reductions were calculated by comparing recovery from UV-C-exposed sites versus untreated controls.

For each UV-C experiment, a colorimetric indicator (UVC Dose Indicator, American Ultraviolet, Lebanon, IN) was placed adjacent to the inoculation site to provide a visual assessment of UV-C delivery to the bin.

### Data analysis

There is no standard level of germicidal activity recommended for surfaces. Rutala *et al.* [30] has suggested that disinfectants that demonstrate a 3-log or greater reduction on carriers are likely to be clinically effective on surfaces. Although some investigators require 4-log or greater reduction in viruses as a requirement for virucidal activity [31], others consider a 3-log or greater reduction to be sufficient as an indication of effectiveness of a disinfectant [32–34]. Therefore, for purposes of analysis, we considered a ≥3-log reduction in recovery of organisms inoculated onto the bin surfaces to be effective for decontamination.

Analysis of variance (ANOVA) was performed to compare the mean log_10_ reductions for the different organisms and for different UV-C exposure times. The ANOVA models adjusted for the different surfaces inoculated while assessing the parameters of interest. Least-squares means were considered for contrasts of interest. All analyses were performed using R version 3.5.1 statistical software (The R Foundation for Statistical Computing, Vienna, Austria) and functions from the emmeans package were implemented [35]. *P* values of less than 0.05 were considered significant.

## RESULTS

**Figure 2.**
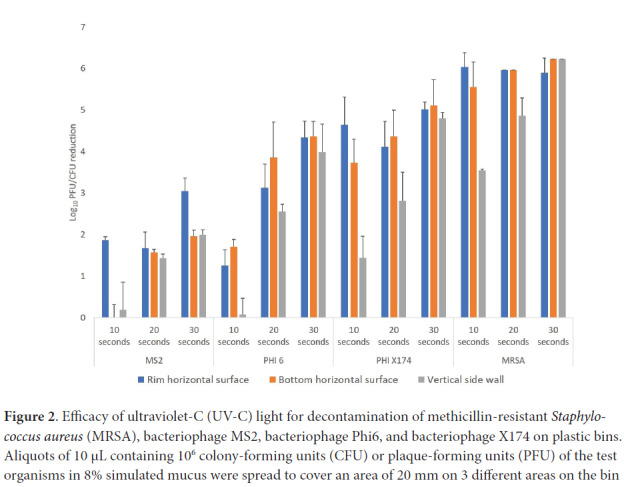
Efficacy of ultraviolet-C (UV-C) light for decontamination of methicillin-resistant *Staphylococcus aureus* (MRSA), bacteriophage MS2, bacteriophage Phi6, and bacteriophage X174 on plastic bins. Aliquots of 10 μL containing 10^6^ colony-forming units (CFU) or plaque-forming units (PFU) of the test organisms in 8% simulated mucus were spread to cover an area of 20 mm on 3 different areas on the bin surface (top rim, horizontal, and vertical) as shown in Figure 1. The bins were exposed to 10-, 20-, or 30-second cycles of UV-C delivered by 2 UV-C lamps positioned horizontally over the bin. Error bars indicate standard error.

Figure 2 shows the log_10_ reductions achieved on the surfaces of the plastic bins with 10-, 20-, and 30-second treatments for the 3 bacteriophages and MRSA. Recovery of MRSA was reduced by >3 log_10_ CFU on all 3 locations on the bin surfaces with all 3 treatment times. Reduction differed significantly across organisms (F = 47.1, df =3, *P*<0.01), and reductions of MRSA were greater than reductions achieved for all the bacteriophages. Reduction also differed significantly with length of treatment (F = 21.8, df =2, *P*<0.01) and, averaging across organisms and sites, reductions with 30-second treatments were significantly greater than reductions with 10- or 20-second treatments (*P*<0.01 for contrasts). Reductions of 3 log_10_ CFU/PFU were achieved with 30-second treatments for all the organisms except bacteriophage MS2 which was reduced by >2 log_10_ at each site with a 30-second treatment. Similar results were obtained when the test organisms were inoculated onto 20-mm steel disks, spread to cover the surface area, and air dried.

**Figure 3.**
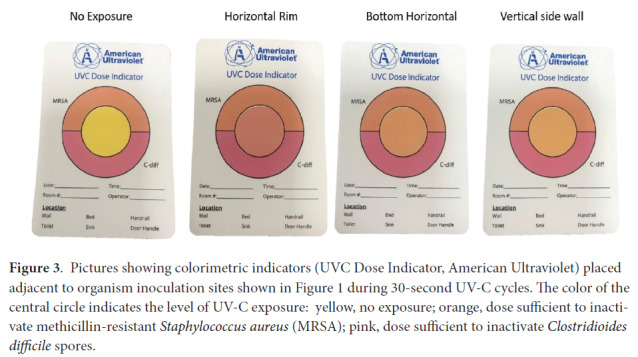
Pictures showing colorimetric indicators (UVC Dose Indicator, American Ultraviolet) placed adjacent to organism inoculation sites shown in Figure 1 during 30-second UV-C cycles. The color of the central circle indicates the level of UV-C exposure: yellow, no exposure; orange, dose sufficient to inactivate methicillin-resistant *Staphylococcus aureus* (MRSA); pink, dose sufficient to inactivate *Clostridioides difficile* spores.

Figure 3 shows changes in the UV-C colorimetric indicators with the 30-second duration UV-C treatment at each inoculum site. The 30-second treatment resulted in a color change from yellow (untreated) to orange indicating delivery of a sufficient dose to reduce MRSA at each test location.

## DISCUSSION

Our results demonstrate that short cycles of UV-C could be effective for decontamination of airport security bins. A 30-second UV-C cycle reduced MRSA and the enveloped bacteriophages Phi6 and Phi X174 by greater than 3 log_10_ and reduced the non-enveloped bacteriophage MS2 by greater than 2 log_10_. The greater than 3-log_10_ reduction met our pre-established criteria for decontamination. Our results are consistent with previous studies that demonstrated substantial reductions in pathogens on surfaces with short UV-C cycles delivered in proximity to the treated surface [21–22, 24–25].

In addition to efficacy, there are several practical issues that make airport security bins an attractive target for application of UV-C technologies. The bins are touched repeatedly by multiple users and by suitcases and personal items in a hurried setting that is not conducive to standard cleaning and disinfection processes. Having passengers use disinfectant wipes before loading bins or spraying bins with liquid disinfectants would not be feasible for repeated applications between each use. In order to apply UV-C to airport security bins, there is a need for a technology that would provide automated movement of bins through a decontamination process with UV-C bulbs positioned on all sides of the bins. One requirement for such a technology would be that the UV-C dosing is delivered in an enclosed space that would prevent external or human bystander exposure to UV-C light. Devices that deliver UV-C in an enclosed space have previously been developed for decontamination of touchscreens, keyboards, and small items such as tablets, cell phones, and pens [24–25, 36–37].

Our study has several limitations. First, we used benign bacteriophages rather than viral pathogens for testing. Further work is needed to evaluate bin decontamination in real-world settings with viral contamination. Second, we only studied one type of bin that was a surrogate for actual security bins. Additional testing with different types of bins is needed. Third, we did not compare the efficacy of UV-C with other technologies or with standard cleaning and disinfection. As noted previously, we anticipate that UV-C is more feasible but not more effective than standard cleaning and disinfection. Finally, we only tested one UV-C cycle. In practice, repeated cycles of UV-C would be delivered after each use of the bins.

In summary, our results suggest that UV-C light could be effective for rapid decontamination of airport security bins. UV-C light is ideally suited for decontamination of hard, smooth surfaces such as security bins that can be positioned close to the light source allowing for short treatment cycles. UV-C light is particularly well-suited for decontamination of items such as security bins that are not readily amenable to standard cleaning and disinfection processes. Thus, UV-C light decontamination merits consideration for security bin decontamination in the setting of concerns regarding transmission of viral respiratory pathogens such as SARS-CoV-2 in airports. Further studies are needed to evaluate the utility of UV-C in real-world settings in airports. There is also a need for development of technologies that would provide automated movement of bins through a UV-C decontamination process. Finally, although the current study focused on security bins, it is acknowledged that similar shared high-touch surfaces such as touchscreens might also be considered for UV-C decontamination.
